# Atypical Histiocyte-Rich Sweet's Syndrome

**DOI:** 10.1155/2017/8150719

**Published:** 2017-10-18

**Authors:** Sharon Chi, Marcia Leung, Mark Carmichael, Michael Royer, Sunghun Cho

**Affiliations:** ^1^Internal Medicine Residency Program, Tripler Army Medical Center, 1 Jarrett White Road, Honolulu, HI 96859, USA; ^2^John A. Burns School of Medicine, University of Hawaii, Honolulu, HI, USA; ^3^Hematology-Oncology Service, Tripler Army Medical Center, Honolulu, HI, USA; ^4^Department of Pathology, Walter Reed National Military Medical Center, Bethesda, MD, USA; ^5^Dermatology Service, Tripler Army Medical Center, Honolulu, HI, USA

## Abstract

Sweet's Syndrome is a rare neutrophilic dermatosis thought to be a result of immune dysregulation occurring in the setting of drug exposure, recent infection, pregnancy, and underlying malignancy or idiopathic with specific and widely accepted diagnostic criteria established in the literature. Other organ systems can be involved with varying degrees of severity. An unusual case of Sweet's Syndrome associated with myopericarditis, acral involvement, and atypical histological findings with predominance of histiocytes is described here.

## 1. Introduction

Sweet's Syndrome is characterized by neutrophilic dermal infiltration that is idiopathic (classical Sweet's) or drug-induced or is attributed to a predisposing condition such as malignancy, preceding infection, or pregnancy [1]. Its pathogenesis has been hypothesized as a manifestation of cytokine dysregulation or autoimmunity [1]. In addition to the skin, other organs can be involved, including the heart on rare occasions [2, 3].

The widely used diagnostic criteria for Sweet's Syndrome include the presence of the two major criteria (acute painful erythematous plaques or nodules and dense neutrophilic infiltrate without vasculitis on histological evaluation) and four minor criteria (fever, associated condition such as malignancy, response to steroid or potassium iodide treatment, and lab abnormalities); both major criteria and at least two minor criteria are required for a diagnosis to be made [4]. However, several histologic and clinical variants exist, including histiocytoid and acral Sweet's Syndrome [5]. Furthermore, there are previously published reports of lymphocyte and histiocyte abundance in cases of Sweet's Syndrome [6–9]. A description of an unusual case of Sweet's Syndrome associated with myopericarditis, acral involvement, and atypical histiocyte-rich histologic findings on skin biopsy is presented herein. Informed consent was obtained from the patient for publication of his case.

## 2. Case Presentation

**Figure 1:**
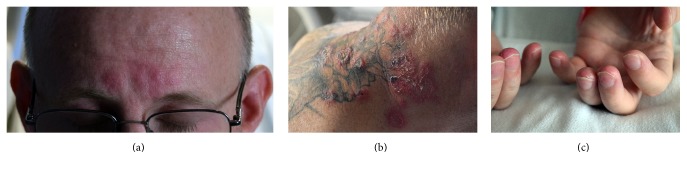
*Skin findings*. (a) Edematous papules and plaques of acute lesions of Sweet's Syndrome. (b) Well-established lesions of Sweet's Syndrome consisting of annual papules and plaques with scale and crust. (c) Finger changes seen in our patient with subungual inflammation splinter hemorrhages and inflammation and scaling of several fingertips.

A 41-year-old previously healthy nonsmoking male was admitted for non-ST-segment-elevation myocardial infarction after sudden substernal chest pain. Three weeks earlier, bilateral knee, elbow, wrist, and hand pain with multiple erythematous papules and plaques on his neck, forehead, and forearms abruptly developed without response to naproxen (Figure 1). He did not take any other medications. Painful erythema and edema of the fingertips with splinter hemorrhages of the nails appeared at two weeks (Figure 1). Review of systems revealed night sweats, nausea, and nonbloody diarrhea three days before initial presentation. Lab studies revealed a troponin I level of 10.2 ng/mL (0–0.034 ng/mL), WBC count of 15.05 × 10^3^/*µ*L (4.4–9.4 × 10^3^/*µ*L) with elevated neutrophils, elevated hepatic enzymes, ESR of 84 mm/h (0–15 mm/h), and CRP of 524 mg/L (<10.0 mg/L). Coronary angiography was unremarkable. Cardiac MRI revealed myopericarditis.

**Figure 2:**
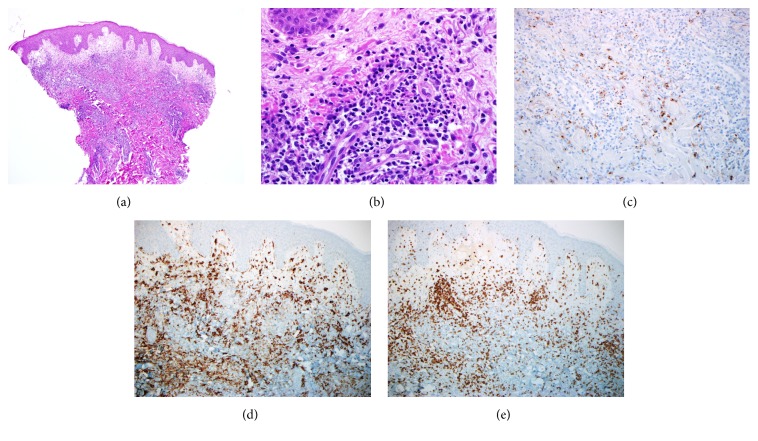
*Biopsy findings*. (a) Histological section showing fairly prominent papillary dermal edema and a somewhat dense dermal infiltrate comprised of mixed inflammatory cells (hematoxylin-eosin, original magnification: ×40). (b) Many large histiocytic cells are present as well as lymphocytes and scattered neutrophils (hematoxylin-eosin, original magnification: ×400). Leukocytoclastic debris is noted, but evidence of vasculitis is lacking. (c) Myeloperoxidase immunohistochemical stain reveals scattered neutrophils but is negative in the histiocytic cells (original magnification: ×200). (d) Histiocytic cells are confirmed to be histiocytes and not mononuclear myelocytes (CD163 immunohistochemical stain, original magnification: ×100). (e) Background T lymphocytes (CD3 immunohistochemical stain, original magnification: ×100).

A diagnosis of Sweet's Syndrome was suspected based on clinical presentation and skin findings. Skin biopsy was performed and systemic steroids were initiated. The skin lesions and joint pain significantly improved within 24 hours of high-dose 1 mg/kg prednisone treatment. Analysis of the skin biopsy from a 22-day-old lesion is shown in Figure 2. A 30-day course of prednisone plus colchicine was prescribed, followed by an aspirin taper. Extensive workup for infectious, rheumatological, and malignant associations was unrevealing and included multiple blood cultures, viral studies, imaging, esophagogastroduodenoscopy, and colonoscopy.

## 3. Discussion

The diagnosis of Sweet's Syndrome was initially suspected clinically with abrupt onset of skin lesions, leukocytosis, and elevated inflammatory markers, as well as a preceding viral-like illness. Histological findings of papillary dermal edema, karyorrhexis, and the presence of neutrophils within the inflammatory infiltrate without evidence of leukocytoclastic vasculitis supported Sweet's Syndrome. However, the infiltrate was composed predominantly of histiocytes and lymphocytes. Immunohistochemical analysis did not show clonality. The diagnosis was based on the clinical morphology and distribution of the lesions, acute onset, inflammatory markers, abrupt response to steroids, preceding illness, and supportive biopsy.

Cardiac involvement is rare in Sweet's Syndrome. Various cardiac sequelae are described in the literature including myopericarditis, valvular disease, coronary artery occlusion, and aneurysm [1–3]. In this case, high-dose prednisone therapy was initiated early due to concern for worsening myopericarditis. A course of colchicine plus prednisone was followed by high-dose aspirin and colchicine due to concern for myopericarditis relapse with glucocorticoid monotherapy.

Another unique feature was fingertip and nail involvement (Figure 1(c)). Neutrophilic dermatosis of the hand is a variant of Sweet's Syndrome with skin lesions isolated to the hands [5]. In our patient, hand involvement occurred two weeks after initial symptoms.

This case illustrates the presence of atypical histological findings in a case of Sweet's Syndrome associated with myopericarditis and acral involvement. These findings challenge the currently accepted diagnostic criteria for Sweet's Syndrome based on the predominance of histiocytes and lymphocytes over neutrophils. This is in line with prior observations also noting a predominance of histiocytic infiltrates in established lesions of Sweet's Syndrome.
